# The Beneficial Role of Retinoids in Glomerular Disease

**DOI:** 10.3389/fmed.2015.00016

**Published:** 2015-03-23

**Authors:** Sandeep K. Mallipattu, John Cijiang He

**Affiliations:** ^1^Division of Nephrology, Department of Medicine, Stony Brook University, New York, NY, USA; ^2^Division of Nephrology, Department of Medicine, Icahn School of Medicine at Mount Sinai, New York, NY, USA; ^3^Renal Section, James J. Peters VA Medical Center, New York, NY, USA

**Keywords:** retinoic acid, retinoic acid receptor alpha, podocytes, FSGS, HIVAN, retinol-binding proteins

## Abstract

The primary etiology of CKD is a direct consequence of initial dysfunction and injury of the glomerulus, the main filtration system. Podocytes are terminally differentiated epithelial cells in the glomerulus, whose major function is the maintenance of this renal filtration barrier. Podocyte injury is implicated in many glomerular diseases including focal segmental glomerular sclerosis and HIV-associated nephropathy. In many of these diseased conditions, the podocyte can either undergo dedifferentiation and proliferation, apoptosis, or cell detachment. Regardless of the initial type of injury, the podocyte ultimately loses its functional capacity to maintain the glomerular filtration barrier. Significant injury resulting in a loss of the podocytes and failure to maintain the renal filtration barrier contributes to progressive kidney disease. Consequently, therapies that prevent podocyte injury and promote their regeneration will have a major clinical impact on glomerular disease. Retinoic acid (RA), which is a derivative of vitamin A, has many cellular functions including induction of cell differentiation, regulation of apoptosis, and inhibition of inflammation and proliferation. RA is required for kidney development and is essential for cellular differentiation in the setting of podocyte injury. The mechanism by which RA directs its beneficial effects is multifactorial, ranging from its anti-inflammatory and anti-fibrotic effects to a direct effect of upregulating podocyte differentiation markers in the podocyte. The focus of this review is to provide an overview of RA in kidney development and glomerular disease. We also highlight the key mechanism(s) by which RA restores podocyte differentiation markers and ameliorates glomerular disease.

## Introduction

More than three decades have passed since the initial description of retinoids, synthetic derivatives of vitamin A (retinol), in human physiology (1). Vitamin A is a critical component of our diet since it cannot be synthesized by our body. It is essential for various cell processes, including kidney development, epithelial cell differentiation, and inhibiting inflammation and cell proliferation (1–5). The widespread use of vitamin A has been hindered by its toxicity, with therapeutic doses frequently resulting in complications of hypervitaminosis syndrome (1). Consequently, the synthesis of first generation derivatives of retinoids, all-*trans*-retinoic acid (atRA), proved to improve efficacy while minimizing toxicity (1). In the canonical retinoic acid (RA) synthesis pathway, retinol is taken up by retinol-binding protein (RBP) in circulation and transferred intracellularly, where it is initially metabolized to retinal by retinol dehydrogenases (RDHs) and transformed into retinaldehyde by retinaldehyde dehydrogenases (RALDHs), with eventual oxidation to RA (6). Subsequently, RA exerts its effect by binding to cytosolic retinoic acid receptors (RAR), which heterodimerizes with retinoid X receptors (RXR), leading to activation of RA response elements (RARE) on target genes (6). In the past two decades, the diverse and critical actions of RA have been clearly illustrated in several pathological processes, from cancer biology to skin treatment to kidney disease (3–5). Our objective in this mini-review is to demonstrate the essential role of retinoids in kidney disease and development as well as its therapeutic benefits in experimental models of glomerular disease. Furthermore, we hope to highlight the deep dichotomy between the numerous basic science studies and the relative dearth of clinical studies on the therapeutic benefits of retinoids in kidney disease.

## Retinoic Acid in Kidney Development

During fetal life, the final nephron number is determined by the branching of the ureteric bud to develop the complete renal collecting system. Studies have clearly demonstrated that RA signaling is critical to this branching nephrogenesis and the final nephron number during kidney development (7–9). This arborization of the ureteric bud is specifically regulated by RA signaling from the stromal mesenchyme (9). Batourina et al. demonstrated that RA from the stromal mesenchyme regulates the expression of a tyrosine kinase receptor, *Ret*, in the ureteric bud during kidney development (9). *Ret* is a proto-oncogene that encodes for a tyrosine kinase receptor and is expressed from the initial stages of the Wolffian duct through the development of ureteric bud. Upon stimulation from RA, the epithelial cells with *Ret* expression initiate the outgrowth and subsequent branching of the ureteric bud (10). Impaired RA signaling directly limits the ureteric bud branching, thereby hindering the development of the collecting system (9). In addition, the authors observed that the deletion of *Rar*α and *Rar*β*2* (isoforms of receptors for RA) in the stromal mesenchyme downregulated the expression of *Ret* in the ureteric bud and impaired the ureteric bud outgrowth in mice (8, 9). Furthermore, others have confirmed that the expression of these receptors (*Rar*α and *Rar*β*2*) is localized to the cells in the stromal mesenchyme, rather than in the cells of the developing nephron (8). Consequently, compound mutations in *Rar*α and *Rar*β*2* cause the stromal precursor cells to remain along the periphery, with few cells between the collecting ducts in the developing medullary zone (8). These mutations also contribute to the downregulation of *Ret* in the ureteric bud, thereby resulting in limited ureteric bud branching and small kidney size at birth (8). Thus, this paracrine-signaling pathway between RA and *Ret* expression is required for branching morphogenesis and the development of the renal collecting system.

Since RA is essential for branching morphogenesis and contributes to the final nephron number, the altered expression of genes involved in RA metabolism has been associated with impaired kidney development (11). For instance, mice lacking in *Raldh2*, enzyme required for the irreversible oxidation of retinaldehyde to RA, are embryonically lethal due to low RA levels and failed organogenesis (12, 13). Conversely, the authors observed that homozygosity for a common variant within the promoter region of the gene encoding for *RALDH2*, conferred an upregulation in *RALDH2* with a subsequent increase in plasma RA levels and kidney volume in humans. The authors suggest that the presence of this variant may especially be protective in individuals that are nutritionally deficient for vitamin A during kidney development by maintaining RA signaling (11). Combined, these studies reveal the essential and protective role of RA signaling in kidney development.

## Retinoic Acid in Glomerular Disease

Other than the critical role that RA plays in kidney development, RA has been demonstrated to restore differentiation markers in cellular injury as well as induce the differentiation of kidney progenitor cells. Furthermore, RA has also been shown to attenuate inflammation and apoptosis in models of podocyte injury. Finally, the progression in podocyte injury has been closely linked to RA metabolism.

### Retinoic acid signaling in podocyte differentiation

**Figure 1:**
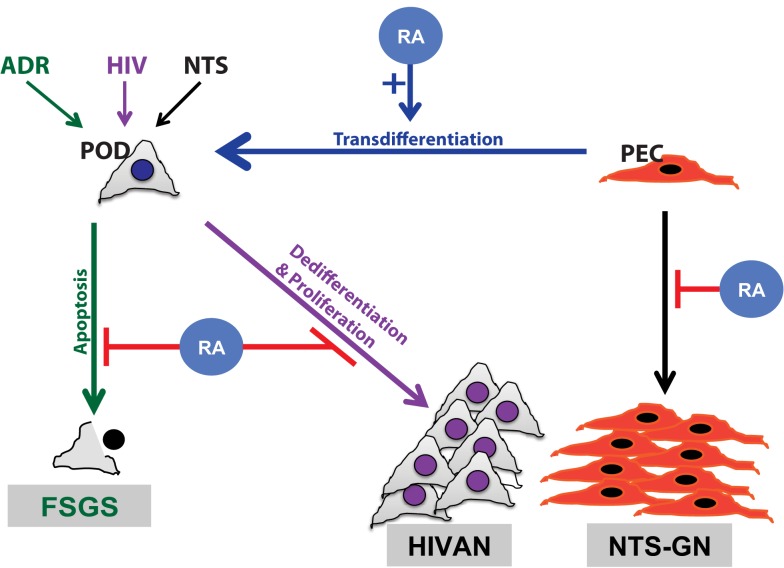
**Schematic diagram of retinoids in podocyte injury**. Retinoic acid (RA) has been demonstrated to play a critical role in attenuation of podocyte injury. The mechanism by which RA abrogates podocyte injury is dependent on the type of inciting injury. Podocyte apoptosis is improved with RA treatment in a murine model of adriamycin-induced nephropathy, focal segmental glomerulosclerosis (FSGS) model (green). Podocyte dedifferentiation is attenuated with RA treatment in models of HIV-associated nephropathy (HIVAN) (purple). In a murine model of crescentic glomerulonephritis, using nephrotoxic serum (NTS), RA treatment minimized parietal epithelial cell (PEC) proliferation (black) and restored podocytes by PEC trans differentiation (blue).

Podocytes are terminally differentiated visceral epithelial cells in the glomerulus, whose function is critical to the maintenance of the glomerular filtration barrier. These highly specialized cells express distinct podocyte differentiation markers and lack the ability to proliferate. Previous studies have illustrated that in the setting of HIV-associated nephropathy (HIVAN) and collapsing focal segmental glomerular sclerosis (FSGS), the podocyte loses its terminal differentiation markers and reenters the cell cycle, leading to cell proliferation (14, 15) (Figure 1). RA has clearly been shown to exhibit anti-proliferative with pro-differentiation effects in multiple tissues, including the kidney and specifically in the podocyte (16–18).

Earlier studies have demonstrated that treatment with RA reduced markers of proliferation and preserves podocyte-specific differentiation markers in *in vitro* and *in vivo* models of HIVAN (17). In models of HIVAN, the expression of HIV-1 transgene induces the podocyte to dedifferentiate and reenter the cell cycle (19). We have demonstrated that treatment with RA abrogates this process in a cyclic adenosine monophosphate (cAMP)-dependent manner (19). For instance, RARα antagonists impede the production of cAMP and the downstream anti-proliferative effects in the podocyte. Similarly, the use of cAMP inhibitors diminished RA-induced podocyte differentiation (19, 20). In contrast, the use of cAMP agonists restored the podocyte differentiation effects of RA. Furthermore, the use of phosphodiesterase-4 inhibitor, increased cAMP levels and enhanced the differentiation effects of RA in cultured human podocytes infected with HIV-1 (19). In addition, the loss of *RAR*α in HIV-1 transgenic (*Tg26*) mice resulted in a significant increase in albuminuria and accelerated glomerulosclerosis (21). Conversely, RARα agonists, such as Am580, have shown to attenuate podocyte dedifferentiation in *in vitro* and *in vivo* models of podocyte injury (21). Interestingly, the concomitant use of phosphodiesterase inhibitor, Roflumilast, with Am580 resulted in a synergistic therapeutic effect on the podocyte (22). Combined, these findings suggest that treatment with RA inhibits podocyte dedifferentiation in a cAMP-dependent manner (18, 20).

Although RA-mediated podocyte differentiation is cAMP-dependent, many podocyte differentiation markers lack RARE or cAMP response element-binding protein (CREB) transcriptional binding sites. To address the mechanism by which RA-cAMP signaling mediates podocyte differentiation, recent microarray studies and comparative promoter analyses were performed (23, 24). These studies revealed that Krüppel-like factor 15 (KLF15) transcriptional binding sites exist on many podocyte-specific genes and may serve as a potential downstream target of CREB (23, 24). In general, KLFs are a subclass of zinc-finger family of DNA-binding transcriptional regulators that are involved in a broad range of cellular processes (i.e., cell differentiation, angiogenesis, erythropoiesis) (25). Specifically, KLF15 is a kidney-enriched zinc-finger binding transcription factor that has been shown to mediate differentiation in various cell types (26). Subsequently, it was demonstrated that KLF15 is downstream of RA and CREB and is a critical mediator of RA-induced podocyte differentiation (24). Furthermore, we observed that the glomerular expression of KLF15 is reduced in kidney biopsies from patients with HIVAN and FSGS as compared to healthy control subjects (24). Combined, these recent studies suggest that KLF15 is required for RA-cAMP-mediated podocyte differentiation.

### Retinoic acid contributes to the generation of glomerular transition cells

In FSGS, podocyte injury is observed with proliferation of epithelial cells in Bowman’s space, a finding which has been defined as a pseudo-crescent (27). For years, it remained unclear if these were truly proliferating parietal epithelial cells or merely podocytes that have lost their differentiation markers and have reentered the cell cycle (28, 29). Regardless of the origin, RA has been demonstrated to be critical to this proliferative process (30). For instance, the mechanism by which RA may regulate the transition of parietal epithelial cells to podocytes has been closely examined in several models of podocyte injury (28–31). For instance, in a rat model of membranous nephropathy (where there is a loss of podocyte differentiation markers), RA was shown to restore podocyte number by increasing the number of epithelial cells expressing podocyte differentiation markers in the glomerulus (30). Specifically, the increase in these epithelial transition cells in the glomerulus colocalized to Paired box gene 2 (PAX2), parietal epithelial cell marker, and Wilms-Tumor 1 (WT1), podocyte-specific marker, thereby suggesting that RA treatment induced the transition of parietal epithelial cells to podocytes (30). Similarly, the authors confirmed this using an experimental model of FSGS to illustrate that the beneficial effect of RA in podocyte regeneration may go beyond a specific glomerular disease (30). Since podocytes are terminally differentiated cells with minimal capacity to self-replicate, restoration of these podocytes in the setting of cellular injury is critical to the recovery of glomerular function. This recent evidence demonstrates that RA may be essential to this regenerative process.

### Impaired retinoid signaling contributes to podocyte injury

In nephrotic range albuminuria, the sequestration of RA by albumin prevents podocyte regeneration in murine models of podocyte injury (31). For instance, it was shown that increasing albuminuria prevents the differentiation of progenitor cells toward a podocyte lineage (31). Specifically, Peired et al. demonstrated that sequestration of RA by increasing albuminuria prevented RARE-mediated regulation of podocyte differentiation markers, which was rescued with administration of RA (31) (Figure 1). In addition, *in vitro* studies revealed that albumin overload impairs podocyte differentiation markers without affecting podocyte survival. The authors also showed that RA-induced podocyte differentiation was attenuated in the setting of increasing albumin concentration. Their findings suggest that this process is mediated via the sequestration of RA by albumin, leading to downregulation of RARE and worsening podocyte injury (31).

Retinaldehyde dehydrogenase 2 is not only critical to the production of RA and kidney development, but RALDH2 levels are significantly upregulated as a consequence of injury to the podocyte (32). Using a rat model of podocytopathy (puromycin treatment), the authors demonstrated that *Raldh2* levels increased with podocyte injury (32). Subsequent RA treatment restored podocyte differentiation markers in the setting of elevated *Raldh2* levels. Conversely, vitamin A deficiency delayed podocyte recovery in this experimental model (32). This study revealed that both the substrates and the enzymes involved in RA signaling are required for podocyte repair after injury (32). Although RALDH2 is critical to RA production, the rate-limiting step of RA synthesis and its eventual downstream effects remains the conversion of retinol to retinal by retinol dehydrogenases (RDHs). Although many RDHs are critical for RA metabolism, *RDH9* was recently demonstrated to rescue podocytes from injury using two murine models of FSGS, HIV-1 transgenic model and adriamycin-induced nephropathy model (33). We demonstrated that the overexpression of *RDH9* in cultured podocytes induced the expression of podocyte-specific differentiation markers. In addition, podocyte-specific overexpression of *RDH9*, in both models of podocyte injury, attenuated glomerular injury and podocyte dedifferentiation in mice (33). Combined, these findings suggest that other than simply using RA for treatment, critical molecules in the RA synthesis pathway may serve as a potential therapeutic in the treatment of glomerular disease.

### Anti-inflammatory effects of retinoic acid

The anti-inflammatory of RA has been demonstrated in multiple animal models of glomerular disease (19, 34). This is not surprising, considering the observed anti-inflammatory effects of RA are well-established in other tissues (1). For instance, *in vitro* and *in vivo* models of diabetic kidney disease revealed that treatment with RA ameliorates podocyte injury. Specifically, the treatment of cultured podocytes with RA reduced the activation of inflammatory pathways with inhibition of monocyte chemotactic protein-1 (MCP-1) synthesis, which is typically increased in diabetic conditions (34). In addition, the authors showed a reduction in proteinuria and inhibition of inflammation in RA-treated diabetic rats (34). Prior studies have also revealed that RA suppresses the transcripts of several pro-inflammatory cytokines and chemokines as well as the recruitment of macrophages (19). In addition, the anti-inflammatory role of RA was more evident with its inhibitory effect in an *in vitro* model of lipopolysaccharide (LPS)-induced macrophage production of IL-12 (35). Using this LPS model, the authors determined that the reduction in IL-12 production by RA was dependent on inhibition of NFκB activity, a potential mechanism by which RA reduced inflammation (35).

Retinoic acid has also been demonstrated to exhibit anti-inflammatory effects in the treatment of primary podocytopathies, such as MCD. Treatment with RA prevented foot process effacement and reversed albuminuria in puromycin aminoglycoside nephropathy (PAN) model, a rat model of minimal change disease (32, 36). In this study, the authors reveal that RA-treated PAN rats exhibited less interstitial mononuclear cell infiltration and reduced expression of fibronectin and MCP-1, which are critical factors for monocyte infiltration (36). RA has been demonstrated to abrogate kidney disease in these murine models of lupus nephritis. For instance, treatment with RA ameliorated kidney injury and prolonged survival in the NZB/W F1 mice, a model of lupus nephritis (37). Also, RA-treated mice exhibited a reduction in glomerular IgG deposits as well a reduction in cytokine expression in the lupus nephritis model (37). These similar effects were observed in another murine model of lupus nephritis, MRL/lpr mice. Specifically, RA treatment reduced albuminuria, improved glomerular lesions, and attenuated chemokine and cytokine expression in the kidney (38). In this model, the authors identified that the renal transforming-growth factor beta (TGFβ) was concurrently elevated with RA treatment, suggesting that TGFβ may potentially mediate the anti-inflammatory effects of RA (38). However, future studies are required to precisely define the mechanism by which TGFβ mediates RA-induced anti-inflammatory effects in the glomerulus. In addition, RA has therapeutic benefits in other models of glomerular disease independent of primary podocytopathies. For instance, treatment with RA reduced crescent formation and improved kidney function in animal models of anti-GBM disease, with a reduction in markers of inflammation and proliferation, such as PCNA, TNF-alpha, IL-1B, PDGF, and C/EBP sigma (16). Regardless of the specific experimental model of glomerular injury, these studies clearly demonstrate that the therapeutic benefits of RA in glomerular disease are at least, in part, mediated by its anti-inflammatory effects.

### Other potential therapeutic benefits of retinoic acid in glomerular disease

In addition to the anti-inflammatory effects of RA, treatment with RA has been demonstrated to reduce apoptosis in murine and cell culture models of podocyte injury (Figure 1). For instance, administration of RA to PAN-treated cultured podocytes resulted in inhibition of apoptosis (36). Furthermore, macrophage-specific loss of RXRα in mice demonstrated reduced clearance of apoptotic cells, resulting in glomerular injury resembling lupus nephritis (39).

Retinoic acid can also play a synergistic therapeutic role with other agents in the treatment of kidney disease. Nephrin, podocyte-specific differentiation marker, expression is induced by RA and activated vitamin D, 1,25-dihydroxyvitamin D3 (40). Interestingly, the synergistic increase in nephrin expression is mediated by selective cooperation between RAR and vitamin D receptor (VDR) in cell culture models (40). Therefore, future studies exploring the concomitant use of both these agents in treating glomerular disease may prove to be efficacious.

Other than the potential use of RA in the treatment of primary podocytopathies, RA may provide some therapeutic benefit in the treatment of other glomerular diseases. In a rat model of mesangial injury, anti-Thy1.1 nephritis, treatment with RA reduced albuminuria and improved glomerular lesions (19, 41). Specifically, administration of RA reduced endothelin-1 and endothelin Type A and B receptor expression levels, which are critical to the progression of renal injury (19). This suggests that a potential alternate mechanism by which RA treatment may abrogate glomerular injury.

### Obstacles in the clinical use of retinoic acid for treating glomerular disease

Retinoids have clearly been demonstrated to have a therapeutic benefit in experimental models of glomerular disease; however, its translation from the bench to the bedside has been hindered by its toxicity. For instance, the strong experimental evidence for the use of RA in kidney disease had prompted the initiation of NIH sponsored open-label randomized clinical trial with the use of Isotretinoin in the treatment of patients with FSGS and collapsing glomerulopathy (NCT00098020) in 2004. However, the recruitment of subjects has proved to be difficult due to the risk of toxicity associated with retinoids.

Toxicity of retinoids includes CNS abnormalities, craniofacial malformation, liver toxicity, hyperlipidemia, reduced spermiogenesis, myalgia, arthralgias, and mucocutaneous side effects (1). To minimize the toxicity and yet retain the therapeutic benefit of RA, we synthesized and demonstrated that a novel derivative of RARα agonist, boronic acid retinoid (BD4), attenuated kidney injury in the HIV-1 transgenic mice. BD4 retains the therapeutic benefit of RA, but with less toxicity due to metabolic oxidation (42). Specifically, we observed that BD4 binds to RARα and exerts its downstream effects with higher affinity and less toxicity as compared to atRA or Am580 (42). Future research is required to identify agents such as these with similar efficacy, but with lower toxicity than atRA. After establishing a safe therapeutic profile, we need to take the next step in implementing these agents in clinical trials.

## Conclusion

In conclusion, we provide a brief overview on the essential role of retinoids in kidney disease. Studies extending for more than three decades have illustrated the therapeutic benefit of RA in models of kidney disease. Despite this large body of evidence, clinical studies demonstrating its therapeutic benefit are lacking. Consequently, there is a dire need for the synthesis of newer generation retinoids as well as novel agents involved in RA metabolism that maintain the therapeutic efficacy of retinoids while minimizing its toxicity.

## Conflict of Interest Statement

The authors declare that the research was conducted in the absence of any commercial or financial relationships that could be construed as a potential conflict of interest.
